# Follicle-Stimulating Hormone Increases the Risk of Postmenopausal Osteoporosis by Stimulating Osteoclast Differentiation

**DOI:** 10.1371/journal.pone.0134986

**Published:** 2015-08-04

**Authors:** Jie Wang, Wenwen Zhang, Chunxiao Yu, Xu Zhang, Haiqing Zhang, Qingbo Guan, Jiajun Zhao, Jin Xu

**Affiliations:** 1 Department of Endocrinology, Affiliated Hospital of Weifang Medical University, Weifang, Shandong Province, China; 2 Department of Endocrinology, Shandong Provincial Hospital affiliated to Shandong University, Jinan, Shandong Province, China; 3 Shandong Clinical Medical Center of Endocrinology and Metabolism, Jinan, Shandong Province, China; 4 Institute of Endocrinology and Metabolism, Shandong Academy of Clinical Medicine, Jinan, Shandong Province, China; University of Pecs Medical School, HUNGARY

## Abstract

**Objective:**

The objectives of this study were to observe the changes in follicle-stimulating hormone (FSH) and bone mineral density (BMD) in postmenopausal women, to research the relationship between FSH and postmenopausal osteoporosis, and to observe the effects of FSH on osteoclast differentiation in RAW264.7 cells.

**Methods:**

We analyzed 248 postmenopausal women with normal bone metabolism. A radioimmunoassay (RIA) was used to detect serum FSH, luteinizing hormone (LH), and estradiol (E_2_). Dual-energy X-ray absorptiometry was used to measure forearm BMD. Then, we analyzed the age-related changes in serum FSH, LH and E_2_. Additionally, FSH serum concentrations were compared between a group of postmenopausal women with osteoporosis and a control group. Osteoclasts were induced from RAW264.7 cells *in vitro* by receptor activator of nuclear factor kappa B ligand (RANKL), and these cells were treated with 0, 5, 10, and 20 ng/ml FSH. After the osteoclasts matured, tartrate-resistant acid phosphatase (TRAP) staining was used to identify osteoclasts, and the mRNA expression levels of genes involved in osteoclastic phenotypes and function, such as receptor activator of NF-κB (*Rank*), *Trap*, matrix metalloproteinase-9 (*Mmp-9*) and *Cathepsin K*, were detected in different groups using real-time PCR (polymerase chain reaction).

**Results:**

1. FSH serum concentrations in postmenopausal women with osteoporosis increased notably compared with the control group. 2. RANKL induced RAW264.7 cell differentiation into mature osteoclasts *in vitro*. 3. FSH increased mRNA expression of genes involved in osteoclastic phenotypes and function, such as *Rank*, *Trap*, *Mmp-9* and *Cathepsin K*, in a dose-dependent manner.

**Conclusions:**

The circulating concentration of FSH may play an important role in the acceleration of bone loss in postmenopausal women. FSH increases osteoclastogenesis *in vitro*.

## Introduction

Osteoporosis (OP) is defined as an absolute reduction in the quantity of bone or the atrophy of skeletal tissue. As a global health problem, postmenopausal bone loss is the major determinant of osteoporosis. Postmenopausal osteoporosis (PMOP) is now frequently recognized to occur secondary to alterations in the pituitary-bone axis, whereas reduced estrogen after menopause is viewed as the main pathogenesis [1].

However, numerous studies have found that the rate of bone mass loss during perimenopause is greater than that in postmenopause [2,3], whereas estrogen serum levels during perimenopause are normal [4]. Therefore, estrogen deficiency cannot fully explain the loss of bone density after menopause. A clinical research study found that follicle-stimulating hormone (FSH) levels were strongly associated with bone resorption markers in postmenopausal women [5]. Sun et al. reported that female mice lacking either FSHβ or the FSH receptor (FSHR) were resistant to bone loss despite hypogonadism [6]. These data raise the possibility that FSH causes PMOP independently of estrogen.

To explore the role of FSH in the pathogenesis of postmenopausal osteoporosis, we conducted a cross-sectional study of 248 healthy postmenopausal Chinese women aged over 50 y to measure their serum FSH, luteinizing hormone (LH), estrogen (E2) and bone mineral density (BMD). In addition, the effect of FSH on osteoclast differentiation was examined in the osteoclast cell line RAW264.7.

## Materials and Methods

### Subjects

Between October 2009 and June 2010, postmenopausal women were recruited by the Jinan health organization. The exclusion criteria were as follows: (1) participants with chronic diseases; (2) participants with hepatic disease, renal disease or other endocrine diseases; (3) participants with a history of hysterectomy or bilateral oophorectomy; and (4) participants taking medications, such as glucocorticoids, estrogens, thyroid hormone, bisphosphonate, calcitonin, calcium or active vitamin D analogs. In total, 248 postmenopausal women were enrolled in the present analysis. All participants gave written informed consent by signing the consent forms. The study was approved by the Ethics Committee of the Affiliated Provincial Hospital of Shandong University.

### Methods

#### Sample collection

A questionnaire was administered to each participant and included general information, such as name, ethnicity, date of birth, address, identification card number, educational status, profession, family income, telephone number, history of childbearing, status of medical insurance, involvement in sports, dietary intake, history of smoking and drinking, and family history and personal history of diseases and their treatment. Height and body weight were measured to calculate body mass index (BMI) using the following formula: BMI = body weight (kg)/height (m^2^). Fasting blood samples were drawn for serum FSH, LH and E2 tests.

#### Measurement of blood samples

Serum FSH, LH and E2 concentrations were determined by radioimmunoassay (RIA) (Tianjin Nine Tripods Medical and Bioengineering Colt., Tianjin, China) in the Clinical Laboratory of Shandong Provincial Hospital affiliated with Shandong University. The intra-and inter-assay coefficients of variance of FSH were 5.5% and 8.7%, respectively. The intra-and inter-assay coefficients of variance of LH were 5.4% and 7.5%, respectively. The intra-and inter-assay coefficients of variance of E2 were 7.7% and 8.9%, respectively.

#### Bone mineral density measurement

BMD was measured at the low-median region of the left forearm, which is not dominant in most Chinese people, by a trained technologist using a dual-energy X-ray absorptiometry (DXA) fan-beam bone densitometer (EXA-3000, Osteosys, Seoul, Korea). Based on the World Health Organization (WHO) diagnostic criteria for osteoporosis, osteoporosis was defined as a BMD T-score < 2.5, whereas a BMD T-score ˃ -1 was considered normal. BMD T-scores between -2.5 and -1 were defined as osteopenia.

#### Cell culture

The RAW264.7 cell line was purchased from the Cell Bank of the Type Culture Collection of the Academy of Sciences (Shanghai, China). The cells were cultured in 6-well culture plates at a density of 1 × 10^5^ cells/well in Dulbecco’s Modified Eagle medium (DMEM, Gibco, USA) containing 10% (v/v) fetal bovine serum (FBS, Biochrom, Germany), 100 U/mL penicillin-streptomycin solution (Sigma-Aldrich, USA) and 2 mM L-glutamine (Sigma-Aldrich, USA) at 37°C in a humidified 5% CO_2_ atmosphere [7]. After an overnight incubation, the cells were treated with 0, 5, 10 or 20 ng/mL FSH (Sigma-Aldrich, USA; Product No. F 4021) in the presence of 50 ng/mL receptor activator of nuclear factor kappa B ligand (RANKL, Sigma-Aldrich, USA) for an additional 7 days. The medium was refreshed every 48 h. Osteoclast differentiation was analyzed by measuring tartrate-resistant acid phosphatase (TRAP) activity. TRAP staining was performed using an acid phosphatase/leukocytes (TRAP) kit (Sigma-Aldrich, USA) according to the manufacturer's protocol. TRAP-positive cells containing three or more nuclei were defined as osteoclast-like cells.

#### Quantitative real-time PCR

Total RNA was extracted using TRIzol (Takara Biotechnology, Dalian, Liaoning, China) according to the manufacturer’s instructions. cDNA was synthesized from 500 ng of total RNA using ReverTra Ace reverse transcriptase (Takara Biotechnology, Dalian, Liaoning, China) and oligo dT primers (Takara Biotechnology, Dalian, Liaoning, China). The following polymerase chain reaction (PCR) primers were used in this study:

**Table:** 

*β-actin*	forward	5′-ACCCAGATCATGTTTGAGAC-3′
	reverse	5′-GTCAGGATCTTCATGAGGTAGT-3′
*Trap*	forward	5′- GACGATGGGCGCTGACTTCA-3′
	reverse	5′-GCGCTTGGAGATCTTAGAGT-3′
*Rank*	forward	5′-TTTGTGGAATTGGGTCAATGAT-3′
	reverse	5′-ACCTCGCTGACCAGTGTGAA-3′
*Cathepsin K*	forward	5′-CTGAAGATGCTTTCCCATATGTGGG-3′
	reverse	5′-GCAGGCGTTGTTCTTATTCCGAGC-3′
*Mmp-9*	forward	5′-CGAGTGGACGCGACCGTAGTTGG-3′
	reverse	5′-CAGGCTTAGAGCCACGACCATGCAG-3′

### Statistical analysis

Statistical tests were performed using SPSS 17.0 (Chicago, IL, USA). All the data were expressed as the means ± SD for continuous variables. One-way analysis of variance (ANOVA) was performed to analyze group measurement data. Two-sided P-values < 0.05 were considered statistically significant.

## Results

### 1. Subject characteristics

We divided the 248 postmenopausal women into two groups. The osteoporosis group included 128 participants, whereas the non-osteoporosis group included 120 participants. Table 1 presents participant characteristics.

**Table 1:** Characteristics of participants in different age groups.

Age(y)	Group	N	Height(cm)	Weight(kg)	BMI(kg/m2)
**50–54**	OP	33	159.9±4.64	63.7±9.65	24.9±3.54
	Non-OP	32	158.4±5.64	59.0±7.94	23.5±2.73
**55–59**	OP	30	158.9±5.21	67.7±8.32	26.8±3.56
	Non-OP	33	160.1±4.39	60.8±7.40	23.8±2.86
**60–64**	OP	28	157.0±5.70	66.8±8.31	27.1±2.64
	Non-OP	31	156.9±5.75	60.6±9.57	24.6±3.25
**≥65**	OP	29	157.4±5.24	69.4±9.24	28.3±4.28
	Non-OP	32	155.8±5.12	63.5±10.69	26.1±3.86

The values are expressed as mean ± SD

BMI: body mass index, OP: osteoporosis, Non-OP: non-osteoporosis

### 2. Inter-relationships among serum FSH, LH, E2 and BMD

With increasing age, serum FSH and LH concentrations appeared to increase before peaking at approximately age sixty and gradually declining in postmenopausal women (Table 2). Serum E2 levels and forearm BMD decreased with increasing age.

**Table 2:** The forearm BMD, serum FSH, LH, E2 levels in different groups.

Age(y)	N	FSH(mIU/ml)	LH(mIU/ml)	E2(pg/ml)	BMD(g/cm2)
**50–54**	65	51.24±6.52	50.5±4.53	11.71±2.51	0.34±0.07
**55–59**	63	57.13±4.26*	54.41±3.71*	8.96±2.59*	0.33±0.07*
**60–64**	59	56.93±6.08*	53.85±4.82*	7.41±2.04*	0.31±0.08*
**≥65**	61	48.75±4.84*	47.03±3.93*	6.25±1.91*	0.31±0.07*

The values are expressed as mean ± SD

FSH: follicle-stimulating hormone, LH: luteinizing hormone, E2: estradiol

* P<0.05 vs. the first age group

### 3. Changes in serum FSH concentration in postmenopausal women with osteoporosis

To investigate the changes in serum FSH in postmenopausal women with osteoporosis, we analyzed variations in FSH and E2 in postmenopausal women with osteoporosis compared with the control group (Table 3).

**Table 3:** Comparison of serum FSH and E2 levels between groups.

Age(y)		FSH(mIU/ml)		E2(pg/ml)	
	N	control group	osteoporosis group	control group	osteoporosis group
**50–54**	65	49.26±6.09	53.28±6.42*	12.93±2.20	10.46±2.19*
**55–59**	63	55.13±4.94	58.95±4.73*	9.73±2.51	8.26±2.49*
**60–64**	59	54.96±5.69	58.70±5.96*	8.06±2.15	6.81±1.77*
**≥65**	61	47.22±4.32	50.14±4.92*	6.45±2.02	6.06±1.81

The values are expressed as mean ± SD

* P<0.05 vs. the same age control group

Serum FSH levels were significantly increased, while E2 decreased, in postmenopausal women with osteoporosis compared with the age-controlled group (P < 0.05). A negative correlation between forearm BMD and FSH serum concentrations was noted. This result suggests that serum FSH is closely correlated with the occurrence of osteoporosis in postmenopausal women.

### 4. Differentiation of RAW264.7 cells into osteoclast-like cells

**Fig 1:**
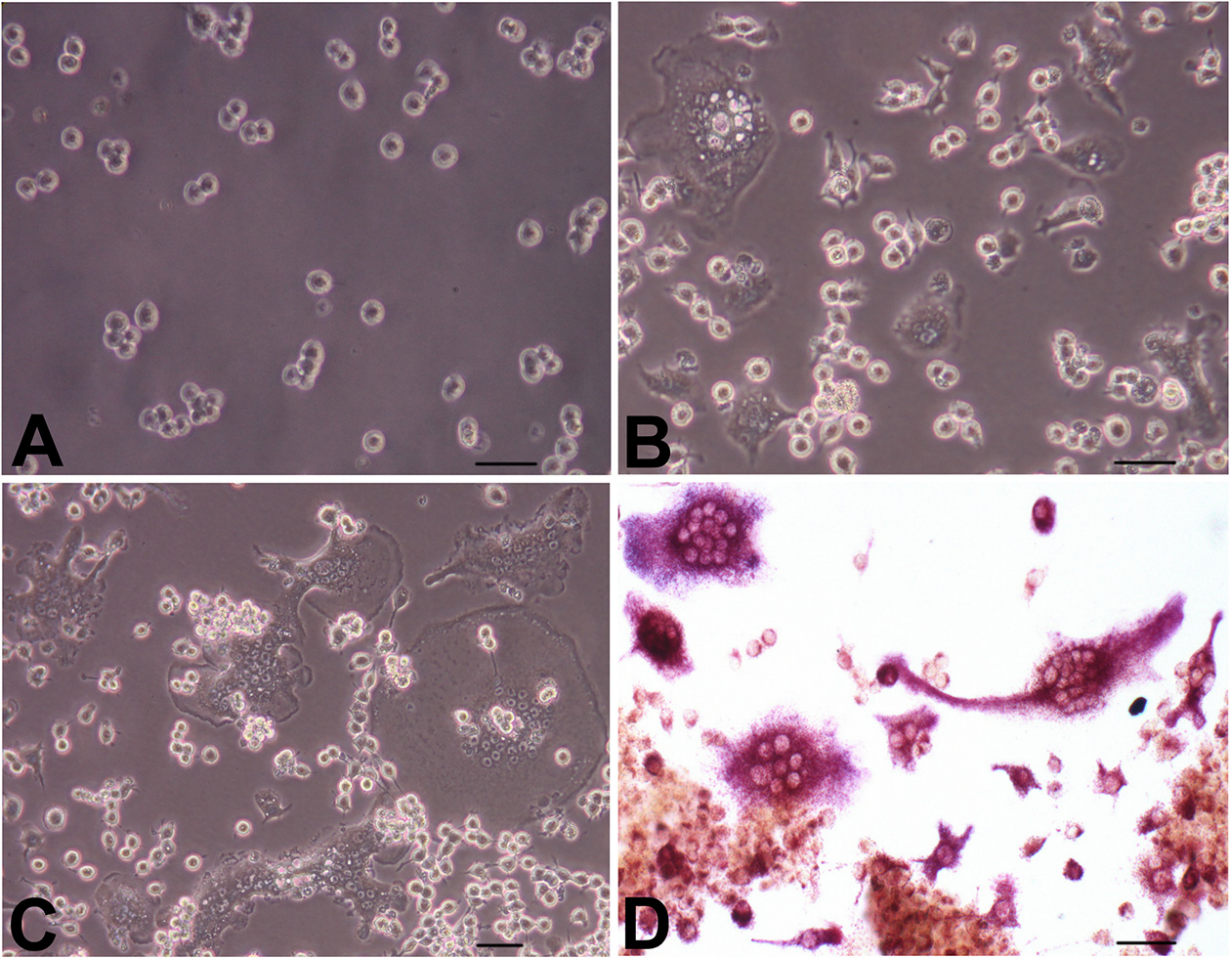
Osteoclast differentiation of RAW264.7 cells induced by RANKL. RAW264.7 cells were induced to differentiate into osteoclasts as described in the Materials and Methods section, for 1, 4 and 7 days. (A, B, and C) Morphological changes in the cells were observed under a light microscope (original magnification 100x). (D) Representative images of TRAP staining (original magnification 100x). Bars: 50 μm.

An inverted phase contrast microscope was used to inspect morphological changes in RAW264.7 cells. The cells began to adhere to the bottom of the culture plate after 1 h of culture; the sizes of the cells were the same, and the shape was mostly round and irregular. The cells contained one or two nuclei, and some cells had pseudopodia. When 50 ng/ml RANKL was added to the culture medium, the morphology of the RAW264.7 cells began to change after three days, with some large cells appearing scattered throughout the plates. On the fourth and fifth days, increasing numbers of irregular cells appeared. In addition, the volume of the cells increased, and polynuclear cells appeared; these were the osteoclast-like cells. The number of osteoclast-like cells peaked on the seventh day. After day 7, the osteoclasts gradually underwent apoptosis and decreased in number (Fig 1A, 1B and 1C).

RAW264.7 cells were induced and cultured for seven days in Petri dishes, and cell slides were prepared for TRAP staining. Under a light microscope, the osteoclasts were multinuclear. These cells stained positive for TRAP and exhibited silk-like protuberances on the cell surface. The cytoplasm was red, and the nuclei were vacuolated (Fig 1D).

### 5. The expression of phenotypic and functional genes in osteoclasts

**Fig 2:**
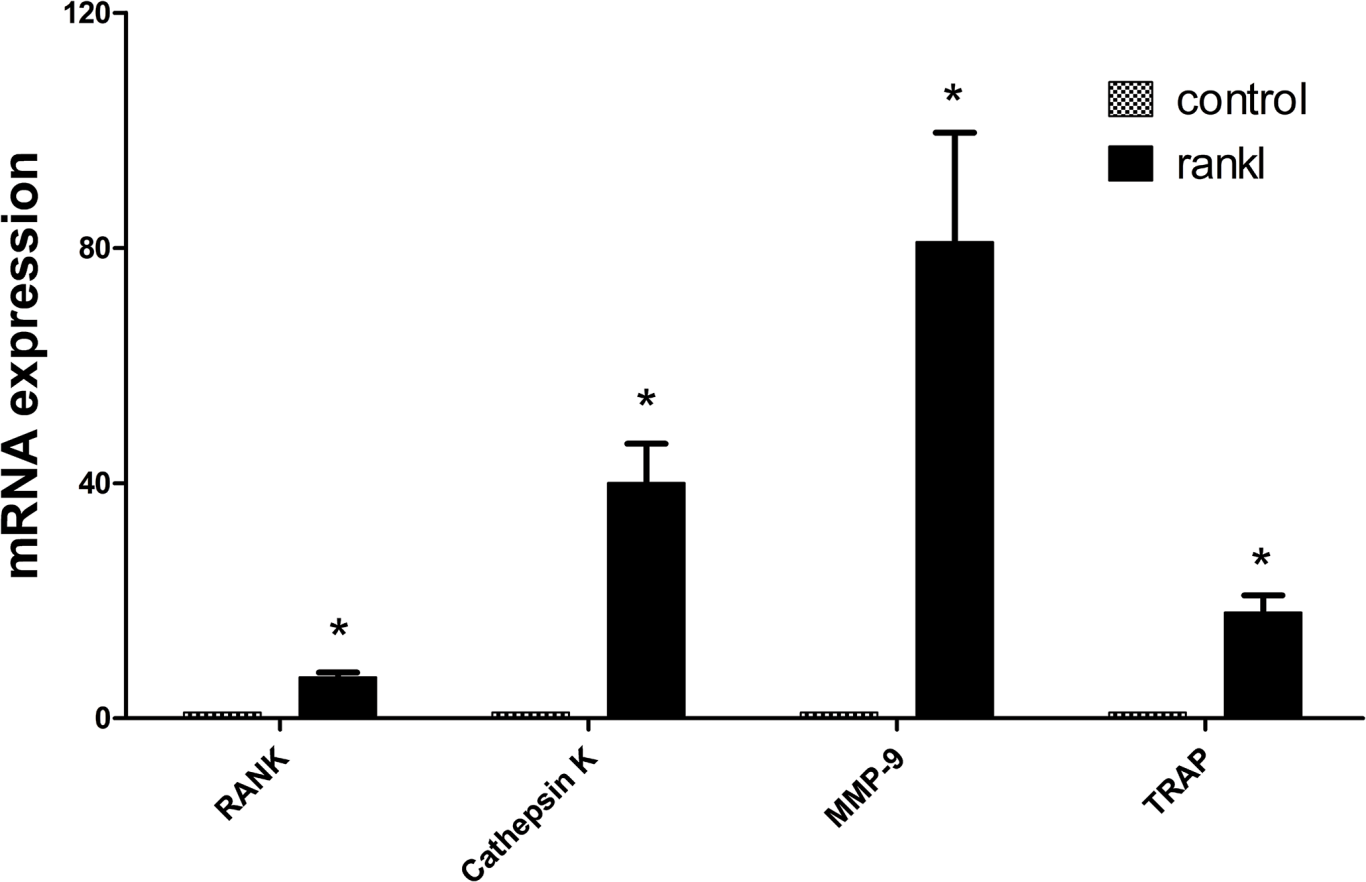
mRNA expression of genes involved in osteoclast phenotypes and function. The mRNA expression levels of *Rank*, *Trap*, *Mmp-9* and *Cathepsin K* were significantly upregulated when the RAW264.7 cells were induced to osteoclasts (*P < 0.05 compared with control).

As shown in Fig 2, low mRNA expression levels of *Rank*, matrix metalloproteinase-9 (*Mmp-9*), *Trap* and the osteoclast functional gene *Cathepsin K* were observed in RAW264.7 cells. The mRNA expression levels of these genes were significantly upregulated when the RAW264.7 cells were induced to differentiate into osteoclasts by the addition of 50 ng/ml RANKL for seven days (P < 0.05). This finding suggests that RAW264.7 cells could be used as an ideal preosteoclast model.

### 6. The effects of FSH on the expression of genes involved in osteoclast phenotypes and function

**Fig 3:**
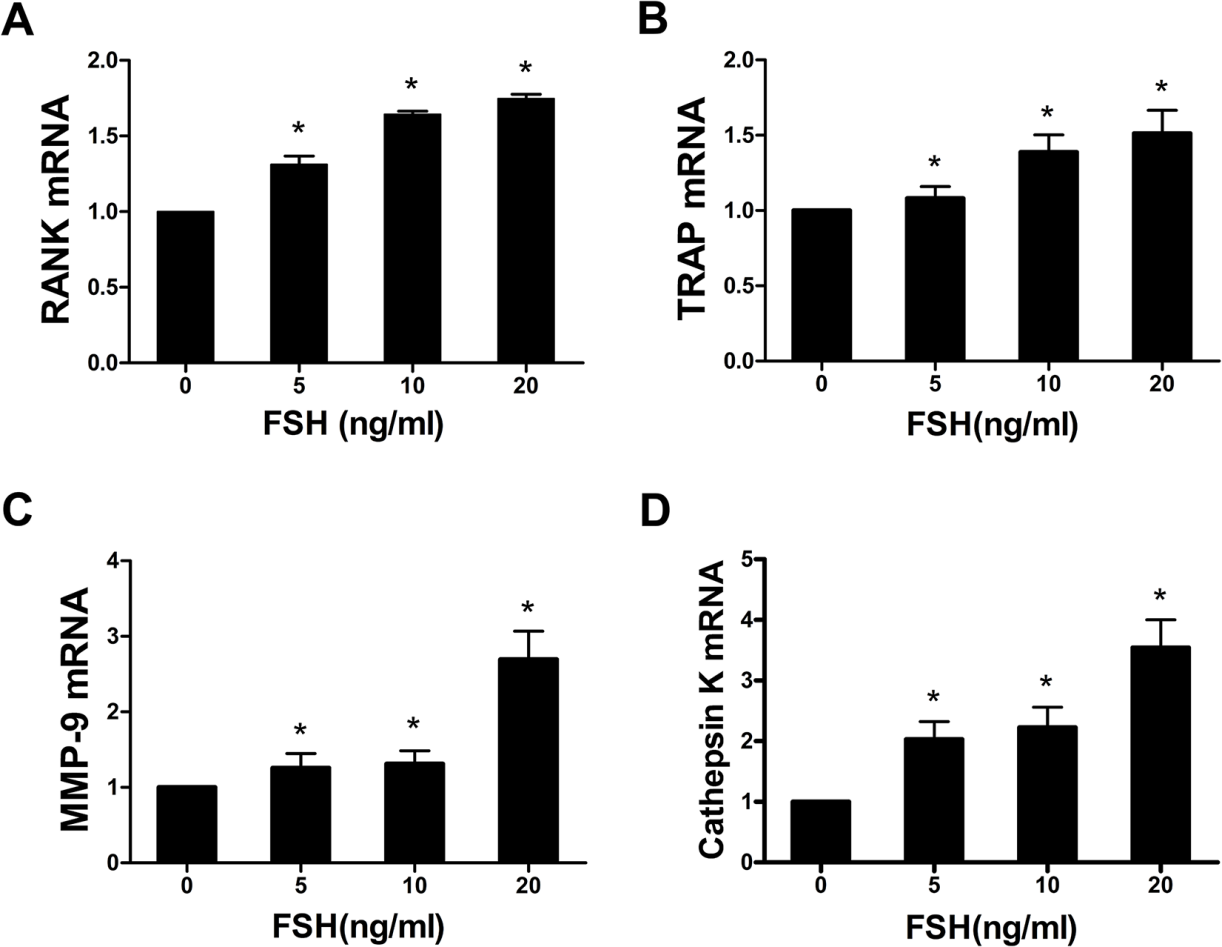
FSH increased *Rank*, *Trap*, *Mmp-9* and *Cathepsin K* mRNA expression in osteoclasts. (*P < 0.05 compared with the control group).

With increased dosages of FSH, *Rank*, *Mmp-9*, and *Trap* mRNA expression levels were gradually increased compared with the control group. The expression of *Rank* mRNA in the groups cultured with 5, 10, and 20 ng/ml FSH increased by 31.3%, 64.4%, and 74.7%, respectively, compared with the control group (P < 0.05). *Mmp-9* mRNA expression increased by 25.6%, 31.1%, and 169.3% (P < 0.05), respectively, and *Trap* mRNA levels increased by 8.13%, 38.7%, and 51.2% (P < 0.05), respectively. These results indicated that the highest mRNA expression levels of the above three genes were obtained with 20 ng/ml FSH. Significant differences FSH in the expression of osteoclast phenotypic genes were observed between 20 ng/ml FSH and 5 ng/ml FSH or 10 ng/ml (P < 0.05). These data implied that FSH upregulates the expression of *Rank*, *Mmp-9*, and *Trap* in a dose-dependent manner (Fig 3A, 3B and 3C).

*Cathepsin K* mRNA expression in osteoclasts was measured using real-time PCR. *Cathepsin K* mRNA levels increased independently of the concentration of FSH compared with the control group. Comparing the 5, 10, and 20 ng/ml FSH treatment groups with the control, *Cathepsin K* mRNA levels increased by 103.2%, 122.6% and 254.2%, respectively. Differences between the 20 ng/ml FSH group and the 5 ng/ml FSH and 10 ng/ml FSH groups were statistically significant (P < 0.05). The highest *Cathepsin K* mRNA levels were observed with 20 ng/ml FSH. Our study suggests that FSH could increase the dose-dependent mRNA expression of the osteoclast functional gene *Cathepsin K* (Fig 3D).

## Discussion

In the present study, postmenopausal women, especially those with osteoporosis, exhibited increased concentrations of FSH. Serum FSH potentially upregulates *Rank*, *Mmp-9*, *Trap*, and *Cathepsin K* mRNA expression in mature osteoclasts and plays an important role in osteoclast-mediated bone resorption.

FSH is a glycoprotein hormone that is secreted by the pituitary and is composed of two non-covalent α and β subunits. In females, the physiological function of FSH involves the promotion of endometrial growth, ovulation, and the stimulation of follicular development. A recent study reported that FSHR gene polymorphisms are associated with bone mineral density and bone turnover in postmenopausal women. Women with the AA rs6166 allele are at higher risk of postmenopausal osteoporosis compared with women with the GG rs6166 allele [8]. *In vitro*, the FSHR is found in osteoclasts; monoclonal or polyclonal antibodies to FSH inhibited the osteoclast formation induced by FSH to an extent similar to that noted in FSHR knockout cells[9]. FSH binds to the FSHR and inhibits the α subunit of G-protein to activate the signaling proteins associated with cell proliferation, ultimately stimulating the formation of osteoclasts and bone resorption.

In our study, serum FSH and LH levels increased until peaking at approximately age 60 y in postmenopausal women; these levels decreased after age 60 y as bone mineral density decreased. This result is similar to previous findings [10]. In addition, the present study found that serum FSH was significantly increased in women with postmenopausal osteoporosis compared with women of the same age with no osteoporosis. This result implied that FSH may be associated with postmenopausal osteoporosis; however, FSH is not the sole cause. The increase in FSH during perimenopause, in which serum estrogen does not decline, may cause the loss of bone mass, whereas estrogen deficiencies may play the dominant role in bone loss in the later postmenopausal period.

FSH is an independent predictor of bone loss. In a previous study, bone mass is significantly decreased in patients with amenorrhea and increased serum FSH levels [11]. The Study of Women Across Nations (SWAN) indicated strong correlations between serum FSH levels and bone loss [12]. FSH serum levels are negatively correlated with BMD: women in the highest quartile of baseline FSH (> 40 IU/l) lost bone 1.-3 to 2.3-fold faster compared with women in the lowest quartile (< 5.8 IU/l) [13]. The results of our study are also consistent with data from the NHANES (National Health and Nutrition Examination Survey) III, a national survey of Americans. In postmenopausal women aged 42 to 60 y with low BMI, an incremental increase in FSH was associated with a 2.78-fold increase in the risk of osteoporosis [14].

However, different researchers, such as Gourlay et al. [15], have reported that serum FSH levels correlated with lean mass but were not associated with bone mineral density in younger postmenopausal women. Thus, greater sample sizes and further long-term studies should be conducted to help elucidate this subject.

E2 deficiency is the dominant cause of bone loss in ovariectomy (OVX) rats [16], but FSH may be closely related to hypogonadal bone loss. A recent study found that FSH can aggravate alveolar bone loss by FSHR independently of estrogen [17]. FSH inhibitors prevent bone loss in ovariectomized rats [18]. The FSH inhibitor leuprorelin (LE) significantly decreased the alveolar bone loss area and osteoclast occurrence compared with non-LE-treated ovariectomized rats [19]. Immunizing ovariectomized rats with the GST- FSHβ antigen significantly prevented trabecular bone loss and thereby enhanced bone strength [20].

FSHR knockout mice exhibited severe hypogonadism, and FSH levels increased sharply in parallel; however, their bone mass was normal [21]. Serum estrogen levels of mice with a knockout of one receptor (FSHβ^+/-^) were normal, and their bone mass was increased compared with wild-type control mice. However, the serum levels of the bone resorption factors TRAP and cathepsin K decreased, whereas bone formation factors were not altered. Moreover, in FSHR knockout mice with normal concentrations of estrogen, bone mass was maintained due to a decline in bone resorption. These studies demonstrated that elevated FSH is closely related to hypogonadal bone loss and direct affects osteoclasts [6].

Clinical studies have demonstrated a strong association between serum FSH and markers of bone resorption [22,23,24]. However, as Table 3 shows higher E2 levels in the control group, we cannot draw strong conclusions about the contributions of the two variables—FSH and E2—to OP. For instance, it is possible that pituitary FSH modulate osteoclast development and thereby influences bone turnover [19] without E2. Therefore, we further examined the effect of FSH on osteoclast differentiation using the RAW264.7 cell line [25,26,27]. These cells differentiate into mature osteoclasts upon induction by RANKL, the key cytokine responsible for osteoclast differentiation [28]. Our study demonstrated that FSH increases the mRNA expression levels of *Rank*, *Mmp-9*, *Trap* and *Cathepsin K* in osteoclasts. FSH may directly affect the differentiation and maturity of osteoclasts and may promote bone absorption.

Iqbal et al. reported that FSH increases TNF-α production in immune cells, stimulates the formation of osteoclasts and bone absorption and modifies bone mass independently of serum estrogen levels [29,30,31]. Short-term treatment with FSH and hCG altered signaling pathways involved in mesenchymal stem cell (MSC) proliferation, including Erk1/2 phosphorylation. These results demonstrate that MSC proliferation is promoted by FSH at menopausal levels [32].

A microarray analysis revealed that electrical stimulation (ES) partially reversed spinal cord injury (SCI)-induced alterations in the expression of genes involved the Wnt, FSH, parathyroid hormone (PTH), oxytocin, and calcineurin/nuclear factor of activated T-cells (NFAT) signaling pathways. ES mitigates SCI-mediated increases in the mRNA levels of the Wnt inhibitors Dickkopf-related protein 1 (DKK1), secreted frizzled-related protein 2 (sFRP2), and sclerostin in *ex vivo*-cultured osteoblasts. These results demonstrate an anti-bone-resorptive activity of muscle contraction by ES that develops rapidly and is independent of the central nervous system (CNS). The pathways involved, particularly Wnt signaling, suggest future strategies to minimize bone loss after immobilization [33].

However, several investigations of genetically manipulated mouse models and clinical data from patients with certain diseases as well as *in vitro* studies have provided inconsistent results. These studies indicate that FSH does not appear to modulate bone mass regulation *in vivo* and does not act directly on osteoclastogenesis *in vitro* [1,34].

FSHRs accelerate bone resorption, whereas estrogen promotes bone formation; these forces are usually balanced. With ovarian failure, low estrogen combined with high FSH causes rapid bone loss. The synthesis of pituitary glycoproteins is noted at distributed sites. This phenomenon is not well studied, but it may further modify the paradigm of central endocrine regulation [35].

Osteoporosis is a common disease in postmenopausal women and is associated with numerous hormones and cytokines. A relationship between FSH and osteoporosis has been observed, but the mechanism is not absolutely clear. In this research, *in vitro* and clinical data demonstrate the effects of FSH on osteoclastic differentiation and function, and FSH levels appear to influence bone loss independently of estrogen concentrations in postmenopausal women. The increase in FSH appears to contribute to increased bone resorption. Our results suggest that FSH measurements could be useful for performing a more comprehensive assessment of bone loss in postmenopausal women.

Moreover, estrogen replacement therapy increases the risk of breast cancer, endometrial cancer and cardiovascular disease, thus making clinicians and patients with postmenopausal osteoporosis concerned about the side effects and risks of this therapy. Our results indicate that a FSH-based vaccine may be a promising therapeutic strategy to reduce bone loss in postmenopausal women.

Due to the complexity of the hormonal changes that occur in perimenopausal and postmenopausal women, further studies are needed, such as *in vivo* animal tests, clinical studies with larger samples and accurate evaluations of signal transduction in osteoclasts.
